# Effects of Sihogayonggolmoryeo-tang (Saikokaryukotsuboreito or Chai-Hu-Jia-Long-Gu-Mu-Li-Tang) for insomnia disorder with prehypertension or stage 1 hypertension: A study protocol for a randomized controlled trial

**DOI:** 10.1097/MD.0000000000020980

**Published:** 2020-07-17

**Authors:** Boram Lee, Yeong-Eun Jeong, Hyo-Ju Park, Young-Eun Choi, Hoseok Kim, Bo-Young Kim, Changsop Yang, In Chul Jung

**Affiliations:** a Clinical Medicine Division, Korea Institute of Oriental Medicine, Yuseong-gu, Daejeon; b Department of Oriental Neuropsychiatry, Daejeon Korean Medicine Hospital of Daejeon University, Seo-gu, Daejeon, Republic of Korea.

**Keywords:** herbal medicine, hypertension, insomnia disorder, Korean Traditional Medicine, Randomized Controlled Trial, Sihogayonggolmoryeo-tang (Saikokaryukotsuboreito, Chai-Hu-Jia-Long-Gu-Mu-Li-Tang)

## Abstract

**Background:**

Insomnia and hypertension are diseases with a high prevalence and a known association with each other. Sihogayonggolmoryeo-tang (SYM) is an herbal medicine traditionally used for the treatment of insomnia disorder concurrent with hypertension. However, no scientific clinical evidence exists supporting the effects of SYM on these disorders. This study aims to explore the feasibility, effectiveness, and safety of SYM for the treatment of insomnia disorder with concurrent prehypertension or stage 1 hypertension.

**Methods:**

A prospective, randomized, wait-list controlled, parallel, pilot clinical trial has been designed for a study to be conducted in Daejeon, Republic of Korea. Thirty insomnia disorder patients with prehypertension or stage 1 hypertension will be randomly assigned to a SYM or wait-list group at a 1:1 ratio. The SYM group will be administered SYM granules twice a day for 4 weeks and followed-up for 2 weeks while the wait-list group will not receive SYM granules. All participants in both groups will be given brochures with instructions for maintaining sleep hygiene and lifestyle modifications to reduce hypertension. Data will be collected at baseline and at 2, 4, and 6 weeks after allocation. The primary outcome is the Insomnia Severity Index score at 4 weeks post-treatment. The secondary outcomes will consist of the Pittsburgh Sleep Quality Index, sleep diary, systolic and diastolic blood pressure, brachial-ankle pulse wave velocity, ankle-brachial index, atherosclerosis biomarkers, the Hospital Anxiety and Depression Scale, the 5-level EuroQol-5 dimensions, and the Patient Global Impression of Change. Adverse events and laboratory test results will be monitored to assess the safety. Data will be recorded in electronic case report forms and analyzed using SPSS Statistics, Version 24.0.

**Discussion:**

This is the first clinical trial to explore the effectiveness and safety of SYM for the treatment of insomnia disorder concurrent with prehypertension or stage 1 hypertension. The results of this study can form the foundation for a future multicenter, large-scale, confirmatory clinical trial.

**Trial registration:**

Clinical Research Information Service, KCT0005001 (registered on May 8, 2020).

## 1 Introduction

According to the 5th edition of the Diagnosis and Statistical Manual of Mental Disorders (DSM-5), insomnia disorder is a quantitative or qualitative sleep disorder, with symptoms including difficulty initiating or maintaining sleep, early awakening, and having sleep difficulties at least 3 times a week for a period of 3 months or longer.^[1]^ The prevalence of insomnia disorder is reported to be 10% to 30% worldwide and as high as 50% to 60% in some cases.^[2]^ Particularly, in the Republic of Korea, according to the disease statistics data from the Korea Health Insurance Review and Assessment Service, the number of patients treated for insomnia has increased continuously from 461,790 in 2014 to 597,529 in 2018.^[3]^ Insomnia not only reduces the health-related quality of life for individuals but also increases the risk of depression, suicide, high blood pressure, and cardiovascular disease.^[4]^ Currently, various pharmacological interventions and cognitive behavioral therapy are being used to treat insomnia; however, their effects on insomnia-associated medical conditions are uncertain. In particular, insomnia is associated with hypertension, and adults with hypertension have a reported 1.5 to 3.18 times higher risk of insomnia than those without hypertension.^[5–8]^ For this reason, treatment of hypertension is considered an ideal secondary target for the treatment of insomnia in primary care settings. Recently, studies on the improvement of blood pressure with cognitive behavioral therapy, which is the primary treatment option for insomnia, have also been conducted.^[9]^

Herbal medicine is an intervention that has been used for centuries in East Asian countries. Several studies have confirmed the effectiveness and safety of herbal medicine for the treatment of insomnia and hypertension.^[10,11]^ In particular, herbal medicine refers to a natural product formulation containing a plurality of active ingredients that act on a plurality of targets and has attracted attention as a promising candidate capable of overcoming the limitations of conventional synthetic drugs with a single active ingredient.^[12]^ Recently, a systematic review of the efficacy and safety of herbal medicine treatment for elderly insomnia patients with hypertension suggested promising possibilities for herbal medicine treatment in improving insomnia, lowering blood pressure, and solving mental health problems such as anxiety and depression.^[13]^

Sihogayonggolmoryeo-tang (SYM; Saikokaryukotsuboreito in Japanese, Chai-Hu-Jia-Long-Gu-Mu-Li-Tang in Chinese) is an herbal prescription comprised of 11 herbs, specifically *Bupleuri Radix, Fossilia Ossis Mastodi, Ostreae Testa, Scutellariae Radix, Cinnamomi Ramulus, Poria Sclerotium, Zingiberis Rhizoma Recens, Zizyphi Fructus, Rhei Radix et Rhizoma, Pinelliae Tuber,* and *Ginseng Radix*. It is frequently used for the treatment of insomnia in East Asia,^[14,15]^ and experimental studies have shown that it can improve sleep disorders by reducing excitability in animal models.^[16]^ The blood pressure of patients with primary hypertension was reported to decrease significantly in 95.98% of cases when SYM was administered.^[17]^ In addition, when SYM combined with antihypertensives was administered to hypertensive patients with depression, both depression and hypertension were significantly improved compared with the results when only antihypertensives were administered.^[18]^ Thus, based on the properties of multiple targets and multiple active ingredients, SYM may affect not only insomnia but also hypertension at the same time and may reduce the complications caused by these disorders. In particular, considering that antihypertensive medications are not recommended in the prehypertensive stage and lifestyle modification is recommended before antihypertensive medications in the first stage of hypertension,^[19,20]^ SYM administration may not only improve sleep quality but also slow the progression of hypertension. However, no study has evaluated the effects of SYM on insomnia disorder concurrent with prehypertension or stage 1 hypertension. Therefore, the aim of this study is to explore the feasibility, preliminary effectiveness, and safety of SYM for the treatment of insomnia disorder with prehypertension or stage 1 hypertension.

## 2 Methods/design

### 2.1 Trial design

**Figure 1:**
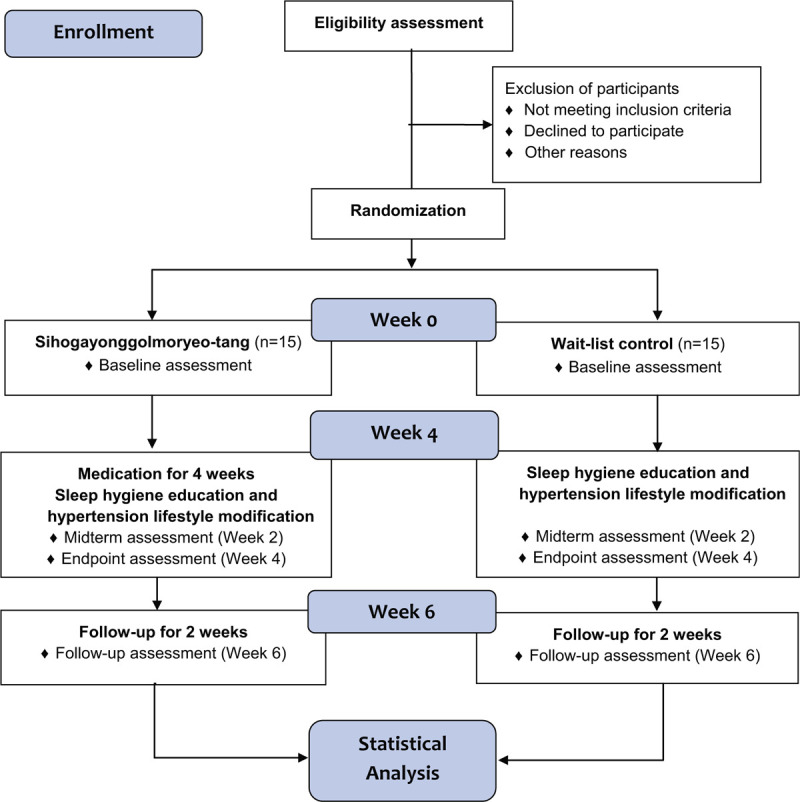
Flow diagram of the trial.

A prospective, randomized, wait-list controlled, parallel, pilot clinical trial will be performed at the Daejeon Korean Medicine Hospital of Daejeon University (Daejeon, Republic of Korea). The trial period will include 4 weeks of medication (twice a day) and 2 weeks of follow-up. The study procedure is summarized in Figure 1 and Table 1. A total of 30 participants will be included and they will be randomly assigned to a SYM group or a wait-list group in a 1:1 allocation ratio. Participants will be informed about the objectives, procedures, and potential benefits, and risks of the trial through standardized interviews prior to their participation and informed consent will be obtained from all participants. Participants will be allowed to withdraw from the study at any time without negative consequences and will be immediately notified when new information regarding the study is obtained. The personal information of the participants will be de-identified and will be accessible only to the investigators involved in this study. This follows the Consolidated Standards of Reporting Trials (CONSORT) Extension for Chinese Herbal Medicine Formulas 2017^[21]^ and Standard Protocol Items: Recommendations for Interventional Trials (SPIRIT) 2013 statement.^[22]^ In addition, the trial will be conducted in accordance with the Declaration of Helsinki and Good Clinical Practice Guidelines.

**Table 1:**
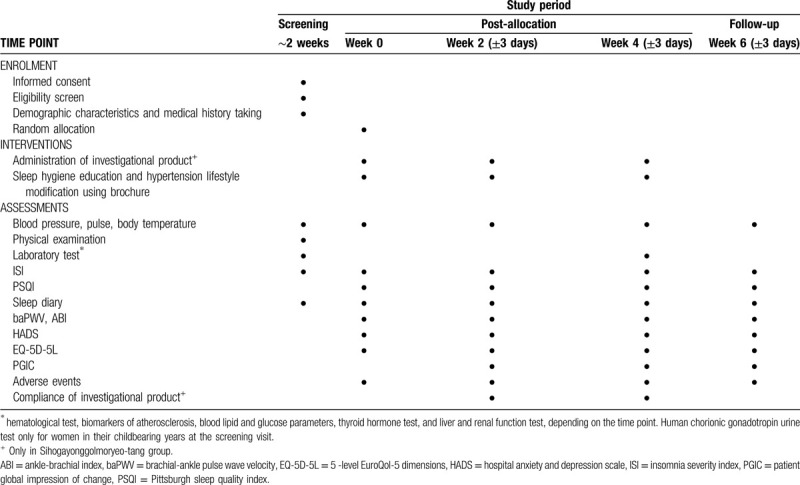
Schedule of enrolment, interventions, and assessments.

### 2.2 Recruitment

Participants will be recruited through offline and online recruitment notices, including posters and webpages. Screening will continue until 30 participants have been enrolled. The estimated recruitment period is from June 2020 to May 2021.

### 2.3 Inclusion criteria

1. adults aged 19 years or over but under 80 years;
2. fulfillment of the DSM-5 diagnostic criteria for insomnia disorder^[1]^;
3. total Insomnia Severity Index (ISI) score ≥15 points;
4. patients with prehypertension (systolic blood pressure [SBP] 120 to 139 mm Hg or diastolic blood pressure [DBP] 80 to 89 mm Hg) or stage 1 hypertension (SBP 140–159 mm Hg or DBP 90–99 mm Hg), as defined in the seventh report of the joint national committee^[20]^;
5. have voluntarily signed written informed consent forms approved by the institutional review board (IRB) after sufficient explanation about this study.

### 2.4 Exclusion criteria

1. started taking sleep medication to improve insomnia or changed the type or dosage of a regularly taken sleep medication within 2 weeks prior to screening;
2. have taken herbal medicine to improve insomnia within 2 weeks prior to screening or have plan to take it to improve insomnia during the study period;
3. started Korean medicine treatment besides herbal medicine (acupuncture, moxibustion, or cupping therapy), dietary supplements, or other non-pharmacological therapies for the purpose of improving insomnia within 2 weeks prior to screening;
4. diagnosed with sleep disorders besides insomnia such as sleep-related breathing disorder, hypersomnia, circadian rhythm sleep disorder, restless leg syndrome, and periodic limb motor disorder;
5. working shifts or changes in day/night work schedule that can impact on circadian rhythm;
6. diagnosed with major depressive disorder, anxiety disorder, panic disorder, or other psychiatric disorders or a Patient Health Questionnaire-9 score ≥ 10;
7. have sleep disturbance caused by any type of pain;
8. uncontrolled thyroid function (abnormal level of free thyroxine (free T4) and thyroid-stimulating hormone (TSH) < 0.1 μIU/ml or TSH > 5.1 μIU/ml);
9. have received antihypertensives, Korean medicine treatment (acupuncture, moxibustion, cupping therapy, or herbal medicine), dietary supplement, or other non-pharmacological therapies for the purpose of controlling blood pressure within 4 weeks prior to screening;
10. a history of aortic constriction, hyperaldosteroneemia, renal artery stenosis, Cushing's disease, chromophiloma, or polycystic kidney disease suspected of secondary hypertension;
11. a history of asthma, chronic obstructive pulmonary disease, cerebrovascular disease (stroke, transient ischemic attack), cardiovascular disease (myocardial infarction, angina pectoris, heart failure, valve disease), malignant tumors, and active tuberculosis within 6 months prior to screening;
12. uncontrolled diabetes mellitus with HbA1c ≥ 9% or fasting blood glucose ≥160 mg/dl;
13. women who are pregnant or lactating, or women who do not agree to use effective methods of contraception during the clinical trial;
14. severe liver or renal disease (aspartate aminotransferase (AST) or alanine aminotransferase (ALT) levels ≥5 times the upper limit of normal or creatinine levels ≥2 times the upper limit of normal);
15. a history of alcohol or drug abuse;
16. genetic problems of galactose intolerance, Lapp lactase deficiency, or glucose-galactose malabsorption;
17. less than 1 month from the end of the intervention in a previous clinical trial;
18. judged to be unsuitable for participation in this clinical trial for other reasons.

### 2.5 Randomization and allocation concealment

A statistician independent of the intervention and evaluation procedures used in the clinical trial will generate random allocation numbers using the statistical program R version 3.6.1 (The R Project for Statistical Computing), with a 1:1 allocation ratio for the SYM group and wait-list group. Participant assignment in this clinical trial will be based on a block randomization method without stratification. Block sizes 2, 3, 5, and 6 will be randomly selected and a sequence that can be assigned to each block size will be randomly selected. This process will be repeated until the target number of participants is reached. The statistician will seal the randomization codes in sequentially numbered opaque envelopes and these envelopes will be kept in double-locked cabinets. Each envelope will be opened by investigators to assign the enrolled participant to the SYM or wait-list group.

### 2.6 Blinding

Due to the study design, the SYM group and the wait-list group, the blinding of participants and prescribing personnel is not possible. However, blinding of the outcome assessors will be maintained during the study period by separating the researchers who will prescribe and explain the investigational product from those who will measure the outcomes. In addition, the statistician who will analyze the results will be blinded to group allocation.

### 2.7 Interventions

Participants in the SYM group will receive 3.0 g SYM granules twice a day before or between meals for 4 weeks. The dosage is based on the regimen approved by the Korea Ministry of Food and Drug Safety. We will use Kracie Sihogayonggolmoryeo-tang Extract Fine Granules manufactured by Kracie Pharma Korea Co., Ltd (Seoul, Republic of Korea) in accordance with good manufacturing practice standards. The SYM granule is a light brown-brown mixture of the hot water extracts of 11 medicinal plants: *Bupleuri Radix* 5.0 g, *Fossilia Ossis Mastodi* 2.5 g, *Ostreae Testa* 2.5 g, *Scutellariae Radix* 2.5 g, *Cinnamomi Ramulus* 3.0 g*, Poria Sclerotium* 3.0 g*, Zingiberis Rhizoma Recens* 0.8 g, *Zizyphi Fructus* 2.5 g*, Rhei Radix et Rhizoma* 1.0 g*, Pinelliae Tuber* 4.0 g, and *Ginseng Radix* 2.5 g per 1 day. A pharmacist will be responsible for the storage, dispensation, and quality control of the SYM granules. If there are any adverse events during the study period, investigators will take appropriate action depending on the severity of the adverse event and the causal relationship with the investigational product.

The wait-list group will not receive SYM granules and will be instructed to maintain conventional care during the study period. The participants in this group will receive 2 weeks of SYM granules as compensatory treatment after the wait-list period if desired under the judgment of Korean medicine doctors, although this will not be included in the analysis. All participants in both groups will be educated and provided with brochures on maintaining sleep hygiene and hypertension lifestyle modification including diet and exercise during the study period.

Concomitant administration of drugs that are judged by the investigator to have no effects on the interpretation of the results of this clinical trial will be permitted. Sleep medication taken regularly from 2 weeks prior to trial participation will be permitted only if the type and dose of the drug are not changed during the clinical trial period. In addition, participants receiving Korean medicine treatment besides herbal medicine, dietary supplements, or other non-pharmacological therapy for the purpose of improving insomnia from 2 weeks prior to trial participation will be permitted if the regimen is not changed during the trial period. However, the administration of herbal medicine for the purpose of improving insomnia will be prohibited during the study period. Participants will be instructed not to change the dose or type of allowed concomitant medications at the screening visit if possible and the investigators will record the details of any changes in case report forms (CRFs).

The use of antihypertensive drugs, central nervous system stimulants, antidepressants, oral steroids, oral contraceptives, and appetite suppressants that can affect sleep or blood pressure will be prohibited during the study period. However, for oral steroids, prednisone or equivalent corticosteroids will be allowed if taken for less than 30 days at a dose less than 0.25 mg/kg/day, which is not considered to affect sleep or blood pressure.^[23,24]^

### 2.8 Outcome measures

#### 2.8.1 Primary outcomes

The primary outcome measure is the total ISI score and will be measured at all visits. The primary endpoint is 4 weeks after treatment. The ISI questionnaire is designed to screen for the diagnosis and severity of insomnia through 7 items corresponding to the symptoms of insomnia. The total score ranges from 0 to 28, with 0–7 indicating no clinically significant insomnia, 8–14 indicating subthreshold insomnia, 15–21 indicating clinical insomnia (moderate severity), and 22–28 indicating clinical insomnia (severe). We will use the validated Korean version of the ISI questionnaire in this study.^[25,26]^

#### 2.8.2 Secondary outcomes

Sleep quality will be measured using the Pittsburgh Sleep Quality Index (PSQI) questionnaire. The PSQI evaluates 7 items including subjective sleep quality, sleep latency, sleep duration, habitual sleep efficiency, sleep disturbances, use of sleep medication, and daytime dysfunction during the past month. A total PSQI score >5 indicates poor sleep quality.^[27]^ The validated Korean version of the PSQI questionnaire will be measured at all visits except for the screening visit.^[28]^ The participants self-reported sleep diary will be collected from 1 week before medication to the end of the trial. The sleep diary will include sleep information such as the bedtime at the previous night, waking time in the morning, sleep latency, and the number and duration of wake times after sleep onset. Sleep diary-derived sleep efficiency will be calculated as (total sleep time/total time in bed X 100%).^[29]^ We will compare the average of sleep parameters contained in the sleep diary during 1 week before each visit.

SBP and DBP will be measured using an automatic blood pressure meter (FT-700R, Jawon Medical Co., Republic of Korea). Blood pressure will be measured at every visit and participants will be instructed not to smoke, drink alcohol, or consume caffeine-containing beverages within 30 minutes before the measurement. At each time point, the participants will be allowed to rest for at least 5 minutes before the first blood pressure measurement. At the screening visit, the blood pressure in both arms will be measured consecutively and the arm with the higher blood pressure level will be measured twice at 1–2-minute intervals. Arms measured consecutively at the screening visit will be continuously measured at other visits, and the average of 3 measurements per visit will be used as an outcome measure. To evaluate the degree of atherosclerosis, the degree of arterial stiffness and stenosis will be assessed noninvasively through the brachial-ankle pulse wave velocity (baPWV) and the ankle-brachial index (ABI) using VP-1000plus (OMRON Healthcare Co., Kyoto, Japan) at all visits except the screening visit.^[30,31]^ In addition, atherosclerosis biomarkers including high-sensitivity C-reactive protein (hs-CRP), erythrocyte sedimentation rate (ESR), homocysteine, and fibrinogen will be measured at the screening visit and 4 weeks after randomization.^[32–35]^

The anxiety and depression levels of participants before and after treatment will be measured using the Korean version of the hospital anxiety and depression scale (HADS) questionnaire.^[36]^ This questionnaire contains 14 items consisting of an odd 7 anxiety subscale and an even 7 depressive subscale, assessed on a 4-point scale (0 to 3). It will be measured at all visits except the screening visit. The health-related quality of life will be measured using the Korean version of the 5-level EuroQol-5 dimensions (EQ-5D-5L).^[37,38]^ This is a self-report questionnaire that evaluates 5 domains including mobility, self-care, usual activities, pain/discomfort, and anxiety/depression using a 5-point scale (1 to 5). The health status will be evaluated using the EuroQoL visual analogue scale (EQ-VAS) drawn with 0–100 vertical lines. In addition, the participants subjective perception of overall improvement after treatment will be assessed using the patient global impression of change (PGIC) questionnaire assessed with a 7-point Likert scale, ranging from “very much worse” to “very much improved”.^[39]^ The time points for each outcome measure are presented in detail in Table 1.

#### 2.8.3 Safety outcomes

Laboratory liver and renal function tests will be conducted on all participants at the screening phase (week 0) and post-treatment phase (week 4) to evaluate the safety of the SYM granules. Vital signs will be examined at each visit. Investigators will conduct causality assessment to all the reported adverse events including subjective or objective symptoms of participants at every visit. All adverse events will be recorded in the CRFs regardless of the relationship with the intervention provided. Investigators will ensure that all participants with adverse reactions receive appropriate medical treatment and follow-up until symptoms are resolved.

#### 2.8.4 Study feasibility outcomes

The recruitment rate (a percentage of the number of enrolled participants relative to the total number of screened participants), adherence rate (a percentage of the number of participants taking 70% of the investigational product relative to the total number of participants), and completion rate (a percentage of the number of participants who complete the trial without dropping out relative to the total number of participants) will be calculated to determine whether a confirmatory, full-scale, randomized controlled trial is feasible.

### 2.9 Sample size

There has been no clinical trial to evaluate the effects of SYM on individuals with both insomnia disorder and prehypertension or stage 1 hypertension. Therefore, a formal sample size calculation was not performed because this study will evaluate the feasibility of subsequent confirmatory randomized controlled trials. With reference to a study that recommends a minimum of 12 participants per group in a pilot study in the biological science field,^[40]^ a total of 30 participants were deemed necessary for each group of 15, anticipating a 20% dropout rate.

### 2.10 Statistical analysis

All statistical analyses will be conducted by an independent statistician using SPSS Statistics for Windows, Version 24.0 (IBM Corp., NY, USA). According to the intention-to-treatment principle, which analyzes all participants who have been randomized, all data obtained from participants who undergo an outcome measurement at least once after randomization will be included in the effectiveness analysis. Per protocol set (PPS) analysis will also be conducted if necessary, including only participants who complete the entire process as described in the protocol with no significant violations. Safety analysis will include all data obtained from participants who take the investigational product at least once.

For each group, categorical data will be presented as frequency and percentage, and continuous data as mean and 95% confidence intervals or median according to the distribution of the data. Any statistical differences between 2 groups will be tested by using the Chi-Squared test or Fisher exact test for categorical data and using the Independent *t* test or Wilcoxon rank sum test for continuous data.

The primary outcome is the total ISI score at 4 weeks post-treatment. A two-sided test with a significance level of 0.5 will be performed using an analysis of covariance with the baseline as the covariate and the treatment group as fixed factors. In addition, the analysis can be performed by setting variables that show statistically significant differences in demographic or social characteristics or variables that can affect insomnia or blood pressure as covariates. The last observation carried forward method will be used for missing data. If necessary, subgroup analysis may be performed by categorizing the baseline characteristics of the participants at the screening visit or the baseline visit. Intra-group changes in the outcome measures from baseline to post-treatment will be analyzed using the Chi-square test or Fisher exact test for categorical data and the paired *t* test or Wilcoxon signed rank test for continuous data. Furthermore, we will use a mixed effect model to identify trend changes.

### 2.11 Data and safety monitoring

A clinical research associate at the Korea Institute of Oriental Medicine (KIOM), a sponsor, will visit the institution regularly to monitor protocol violations, recruitment rate, document reporting, and adverse events during the trial period. The monitoring procedures and schedule will follow the KIOM standard operating procedure. Detected items will be properly resolved through discussions with the investigators. A web-based electronic CRF (Medidata Rave; Medidata Solution Inc., New York, USA) will be used for data collection.

### 2.12 Patient and public involvement

This study is designed to explore the feasibility, preliminary effectiveness, and safety of SYM for treating insomnia disorder concurrent with prehypertension or stage 1 hypertension. SYM is an herbal preparation approved as a generic drug by the Korea Ministry of Food and Drug Safety and is easily accepted by patients. Using SYM for insomnia disorder with hypertension may not only improve insomnia, but also lower blood pressure and prevent polypharmacy, thereby reducing long-term medical expenses. The outcome measures used in this trial are considered important endpoints for patients with insomnia and hypertension in clinical settings. However, patients were not directly involved in developing the study design and will not be involved in the recruitment or conduct of the study.

### 2.13 Ethics and dissemination

The trial protocol has been approved by the IRB of Daejeon Korean Medicine Hospital of Daejeon University (approval number: DJDSKH-20-DR-02) and registered at the Clinical Research Information Service (CRIS) (registration number: KCT0005001). If the protocol requires modification, it will be reapproved by the IRB prior to implementation and documented in CRIS. All participants will receive a detailed description of the research process including the potential benefits and risks, other therapeutic options, and the right to withdraw anytime from a licensed Korean medicine doctor and will be asked to sign an informed consent form prior to participation. The results of this study will be released to the public via publication in a peer-reviewed journal or via conference presentations.

## 3 Discussion

This is a study protocol for a randomized, wait-list controlled, parallel, pilot clinical trial evaluating the effect of SYM on patients with both insomnia disorder and prehypertension or stage 1 hypertension. We will evaluate the preliminary effectiveness and safety of 4-week administration of SYM compared to lifestyle modification only and assess the feasibility of large-scale randomized controlled trials.

Insomnia and high blood pressure are very common diseases worldwide, and the economic burden is substantial.^[2,41]^ In particular, the 2 diseases have been reported to be associated with each other.^[5–8]^ For patients with prehypertension or stage 1 hypertension in particular, active lifestyle modification is recommended before medication.^[19,20]^ If patients with insomnia disorders and prehypertension or stage 1 hypertension taking sleep sedatives have elevated blood pressure and therefore take antihypertensive drugs, there may be risks associated with the combination of multiple drugs. However, there is no recommended conventional medication for treating both insomnia and hypertension.

Herbal medicine contains multiple active components that act on multiple targets and therefore has the potential to overcome the limitations of conventional medication with a single active constituent.^[12]^ SYM is an herbal formula that has been approved by the Korea Ministry of Food and Drug Safety and is commercially marketed for symptoms that accompany hypertension (insomnia, anxiety, and palpitation), nervousness, and morbid night crying of babies. Because SYM is an herbal medicine that is already used for treating patients with insomnia disorder and hypertension, the effectiveness and safety have been proven in historical medical literature with long-term empirical evidence. Our study will provide scientific evidence for the effectiveness and safety of SYM through a clinical trial in accordance with scientific procedures. In addition, SYM can be expected to improve not only insomnia but also blood pressure, which may reduce the risk of polypharmacy in the future.

Although there has been no study specifically evaluating SYM, there are many published studies exploring the efficacy and safety of herbal medicine for insomnia disorder with hypertension. A recent systematic review concluded that herbal medicine may have beneficial effects on insomnia, high blood pressure, and mental health problems for patients with both insomnia disorder and hypertension.^[13]^ However, all included studies were conducted in China, and most studies were not approved by an IRB prior to the trial initiation. In addition, some studies evaluated sleep symptoms using non-validated outcome measures. Therefore, we attempted to improve the quality of existing research by using validated questionnaires as the outcome measures and taking ethical issues into consideration.

The limitations of this study are as follows. First, objective sleep-related outcome measures will not be evaluated in this study. Although polysomnography is considered the gold standard in sleep research, it is not always possible in actual clinical studies because of the associated costs, institutional infrastructure, and patient burden caused by hospitalization for the measurement. In our study, we will not measure polysomnography data for the above reasons, but we will alternatively collect accurate and substantial information related to sleep using ISI and PSQI questionnaires and a sleep diary. In addition, in a previous study, the ISI score was found to be consistent with polysomnography findings.^[42]^ Second, a wait-list control will be used for the control group because creating a placebo granule in the manufacturing company was not possible. Therefore, it will not be possible to maintain the blinding of study participants and prescribing personnel. However, we will do our best to maintain the high quality of the research through the blinding of outcome assessor and statistician.

Nevertheless, to the best of our knowledge, this is the first study evaluating the feasibility and effect of SYM on insomnia patients with prehypertension or stage 1 hypertension using validated questionnaires. Though hypertension itself is not a major problem, it can increase the risk of developing life-threatening cerebrovascular diseases.^[43]^ Insomnia disorder is also known to increase the risk of cardiovascular diseases.^[4]^ Therefore, we will evaluate the degree of atherosclerosis by measuring baPWV, ABI, hs-CRP, ESR, homocysteine, and fibrinogen as markers of cardio-cerebrovascular disease during the study period to investigate the effects of SYM on atherosclerosis. If the treatment effect and safety of SYM compared with the wait-list control is demonstrated through this pilot study, the findings are expected to provide a base for large-scale, confirmatory, multicenter, high-quality randomized control trials to draw definite conclusions regarding the efficacy and safety of SYM for patients with concurrent insomnia disorder and hypertension. Furthermore, the results may provide stronger evidence of SYM as a treatment option for insomnia disorder with hypertension for clinicians, patients, and researchers.

## Author contributions

The protocol was first conceived and designed by BL, with critical contributions from the other authors. BL wrote the first draft of the protocol and YEJ submitted the registration on CRIS. BL, YEJ, HJP, YEC, BYK, CY, and ICJ provided help with the design. HK designed the method for statistical analysis. All authors contributed constructive comments on the manuscript and approved the final paper.
